# An explanatory model of quality of life in high-risk pregnant women in Korea: a structural equation model

**DOI:** 10.4069/kjwhn.2023.11.13.1

**Published:** 2023-12-28

**Authors:** Mihyeon Park, Sukhee Ahn

**Affiliations:** College of Nursing, Chungnam National University, Daejeon, Korea

**Keywords:** High-risk pregnancy, Nursing, Psychological adaptation, Quality of life, Uncertainty

## Abstract

**Purpose:**

This study aimed to develop and validate a structural model for the quality of life (QoL) among high-risk pregnant women, based on Roy’s adaptation model.

**Methods:**

This cross-sectional study collected data from 333 first-time mothers diagnosed with a high-risk pregnancy in two obstetrics and gynecology clinics in Cheonan, Korea, or participating in an online community, between October 20, 2021 and February 20, 2022. Structured questionnaires measured QoL, contextual stimuli (uncertainty), coping (adaptive or maladaptive), and adaptation mode (fatigue, state anxiety, antenatal depression, maternal identity, and marital adjustment).

**Results:**

The mean age of the respondents was 35.29±3.72 years, ranging from 26 to 45 years. The most common high-risk pregnancy diagnosis was gestational diabetes (26.1%). followed by preterm labor (21.6%). QoL was higher than average (18.63±3.80). Above-moderate mean scores were obtained for all domains (psychological/baby, 19.03; socioeconomic, 19.00; relational/spouse-partner, 20.99; relational/family-friends, 19.18; and health and functioning, 16.18). The final model explained 51% of variance in QoL in high-risk pregnant women, with acceptable overall model fit. Adaptation mode (β=–.81, *p*=.034) and maladaptive coping (β=.46 *p*=.043) directly affected QoL, and uncertainty (β=–.21, *p*=.004), adaptive coping (β=.36 *p*=.026), and maladaptive coping (β=–.56 *p*=.023) indirectly affected QoL.

**Conclusion:**

It is essential to develop nursing interventions aimed at enhancing appropriate coping strategies to improve QoL in high-risk pregnant women. By reinforcing adaptive coping strategies and mitigating maladaptive coping, these interventions can contribute to better maternal and fetal outcomes and improve the overall well-being of high-risk pregnant women.

## Introduction

In South Korea (hereafter, Korea), there is a trend towards delayed marriages, resulting in an average age of 33.4 years for first-time mothers in 2021. Furthermore, the percentage of mothers of advanced maternal age, defined as 35 years or older, is 33.8% [1]. A report from the World Health Organization indicates that mothers aged 35 years and above are at a higher risk of developing gestational diabetes mellitus, pregnancy-induced hypertension, and experiencing premature birth, stillbirth, neonatal death, and congenital malformations compared to women aged 20 to 34 years [2]. Alongside the increase in advanced maternal age, there has been a significant rise in the number of pregnancies classified as high-risk over the past decade. Specifically, the number has surged nearly sevenfold, from 27,223 cases in 2009 to 145,868 cases in 2018 [3]. High-risk pregnancies, which pose a threat to the health and life of pregnant women, fetuses, and newborns during pregnancy and childbirth, include factors such as chronic preexisting conditions, advanced maternal age, complications from the current pregnancy, as well as socioeconomic levels, mental health issues, and other considerations [4].

In high-risk pregnancies, 92.2% of women diagnosed with preterm labor require hospitalization for the sake of the fetus’s well-being [5]. Even those who receive outpatient care find it challenging to maintain a stable pregnancy, requiring drug therapy and frequent monitoring. This situation can potentially lead both the woman and her partner to experience maladaptive responses to pregnancy [6]. The often-ambiguous etiology of high-risk pregnancies makes predicting outcomes difficult, and the clarity of treatment results may be compromised [7]. As a result, high-risk pregnant women face heightened uncertainty as their psychological stability is threatened and stress persists due to concerns about the fetus, anxiety over maintaining the unstable pregnancy, fear of miscarriage, and a lack of information [7]. However, specialized education and counseling services for these women are limited. Consequently, they may resort to maladaptive coping behaviors, such as self-blame, rumination, and catastrophizing, in response to negative emotional states and uncertain situations [8].

From a cognitive perspective, coping is recognized as a strategy for regulating emotions, and it is divided into two categories: adaptive coping and maladaptive coping [9]. Adaptive coping aims to decrease uncertainty and psychological distress in pregnant women, thereby improving their mental health and quality of life (QoL). In contrast, maladaptive coping can lead to increased depression and anxiety, which negatively affects QoL [10]. Therefore, it is expected that the selection and implementation of appropriate coping strategies will influence the QoL for high-risk pregnant women by maintaining psychological well-being or managing negative emotions [9]. The factors that influence the QoL for high-risk pregnant women are varied and can have either positive or negative effects. Notable factors that have been reported to significantly impact the QoL for high-risk pregnant women include maternal identity [11], spousal support [12], physical symptoms [13], and depression, anxiety, and fatigue [14]. These factors represent the physical, mental, and social adaptation levels of the pregnant woman. They can be viewed as the emotional and behavioral characteristics of high-risk pregnant women and are suitable for measurement as an adaptation mode that evaluates individual behavior. However, a review of the literature reveals a gap in research on the QoL and influencing factors for high-risk pregnant women diagnosed with various conditions, as most studies tend to focus on pregnant women with no or minimal health issues [15,16]. Current research on the QoL for high-risk pregnant women has often been limited to specific conditions, neglecting the process-oriented and multifaceted aspects of adaptation while emphasizing physical health and emotional states [12]. Therefore, from a nursing perspective, it is crucial to gain a comprehensive understanding of the adaptation and QoL of high-risk pregnant women. Identifying relevant factors will provide evidence for nursing interventions aimed at improving their QoL.

Research on high-risk pregnancies, guided by Roy’s adaptation theory [17], has been reported in two international studies. Amanak et al. [18] examined the influence of this theory on maternal adaptation among women with pregnancy-induced hypertension. Similarly, Widiasih et al. [19] applied nursing plans and interventions based on the adaptation theory to women experiencing premature rupture of membranes and assessed their impact on these women’s physical and psychological well-being. As pregnancy has been suggested to be a series of responses to individual changes and environmental stimuli [20]. Roy’s adaptation model [17] was identified as a suitable theoretical foundation for understanding the QoL of high-risk pregnant women during pregnancy. This theory comprises stimuli, coping mechanisms, adaptation modes, and adaptation. Thus, our model focused on uncertainty, adaptive coping, maladaptive coping, and adaptation modes and the goal of our research was to identify the factors that influence the QoL in high-risk pregnant women. We also aimed to understand the demands related to their QoL. Ultimately, we hope to provide evidence-based data that will help establish intervention strategies to improve the QoL for these women.

### Purpose

The purpose of this study was to construct a hypothetical model explaining QoL in high-risk pregnant women through a literature review of previous studies based on Roy’s adaptation theory [17], to validate the fit between actual data and the model, and to elucidate the direct and indirect relationships among factors. The specific objectives were as follows:

1) To construct a hypothetical model of QoL in high-risk pregnant women.

2) To validate the fit between the hypothetical model and actual data, presenting a model that explains QoL in high-risk pregnant women.

3) To identify the direct and indirect effects, as well as the total effects, among variables influencing QoL in high-risk pregnant women, thereby confirming the causal relationships among variables.

### Conceptual framework and hypothetical model of the study

**Figure 1.**
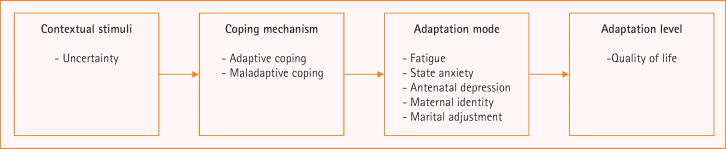
Conceptual framework of quality of life in high-risk pregnant women based on Roy’s model.

The conceptual framework of this study was constructed based on Roy’s adaptation model [17] and a review of the relevant literature. Roy’s adaptation model posits that humans, as psychosocial beings with physical, emotional, and social dimensions, are at the heart of the adaptation system. Individuals utilize this system to respond and adapt to changes in their environment. Roy and Andrews [17] define health as the process of becoming an integrated human being. The ultimate goal of nursing, according to this model, is to promote adaptive processes that enhance the interaction between the human system and the environment. This interaction positively impacts health and QoL. In Roy’s adaptation model, stimuli can be internal or external. The outcomes, based on the stimuli input into the individual’s adaptation system and the level of adaptation, are regulated through behavioral responses via cognator and regulator coping processes. The model identifies four modes of adaptation: the physiological mode, self-concept mode, role function mode, and interdependence mode [17]. These four modes are highly interconnected and act as mediators between the stimuli input into the human system, the coping mechanisms, and adaptation [21]. The experiences of uncertainty, coping, adaptation mode, and adaptation as perceived by high-risk pregnant women can be understood within the context of Roy’s adaptation theory. In other words, this study views pregnancy as an open adaptive system that is constantly interacting with a changing internal and external environment. High-risk pregnancy, characterized by uncertainty, is seen as stimuli input into this system. The study aims to explain the phenomenon of adaptation to pregnancy through the four modes of adaptation—physiological, self-concept, role function, and interdependence—which are altered through coping. The conceptual framework of this study, based on Roy’s adaptation model is shown in Figure 1.

In this study, high-risk pregnancy is considered as a source of contextual stimuli, as women experience uncertain emotions about maintaining pregnancy and fetal well-being due to the diagnosis of complications related to high-risk pregnancy and a lack of specialized information. This uncertainty is input into our framework. We perceive coping mechanisms as cognitive emotion regulation strategies, where regulatory processes help manage emotions and feelings, thereby influencing psychological well-being. Coping during pregnancy is seen as a combination of adaptive and maladaptive coping. This is viewed as a mechanism that influences the cognitive regulatory processes that high-risk pregnant women use to adapt during pregnancy. This adaptation involves physiological factors, emotional factors, and cognitive regulation processes related to roles and interactions with partners. Adaptation involves four modes. In the physiological mode, the primary demand is physiological integration, taking into account the physical and mental fatigue of high-risk pregnant women [22]. The self-concept mode is defined as the integration of beliefs about oneself and psychological symptoms at a given point in time [20]. High-risk pregnant women, compared to low-risk pregnant women, tend to exhibit higher levels of antenatal depression and anxiety related to concerns about maintaining pregnancy and the fetus [23]. Based on the concept definition of high-risk childbearing adaptation [20], emotional factors such as antenatal depression and state anxiety are posited to comprise the self-concept mode. The role function mode focuses on the roles individuals occupy in society. High-risk pregnant women, diagnosed with high-risk pregnancy, may experience negative impacts on the process of integrating their identity as mothers, affecting maternal identity acquisition [24]. Therefore, in this study, we consider the performance of the maternal role and the formation of identity by high-risk pregnant women as factors influencing QoL. The interdependence mode, based on previous research [17], involves behavioral classifications related to interdependent relationships. In this mode, individuals focus on interactions related to affection, respect, and values. High-risk pregnant women, influenced by spousal support and the quality of marital relationships during pregnancy, are expected to impact their QoL. Therefore, we conceptualize marital adjustment as the interdependence mode. Considering the interrelated nature of these five concepts based on the literature review, we incorporate them into the concept of adaptation used in the model. We define the adaptation level as the QoL to which high-risk pregnant women adapt during pregnancy.

**Figure 2.**
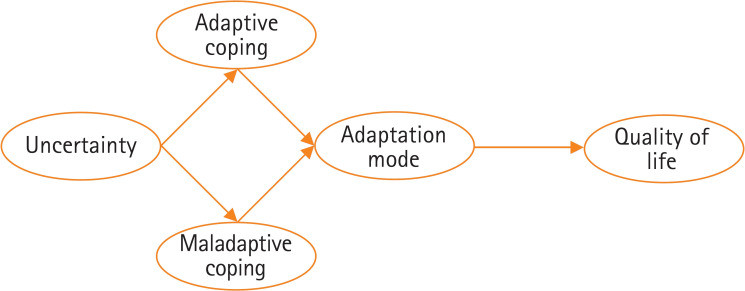
Theoretical model of quality of life in high-risk pregnant women.

Thus, this model focuses on the QoL in high-risk pregnant women. Uncertainty in high-risk pregnant women is established as an exogenous variable, while adaptive coping, maladaptive coping, adaptation mode, and QoL are designated as endogenous variables. The hypothetical model that considers the relationships between these concepts is presented in Figure 2.

## Methods

**Ethics statement:** This study was approved by the Institutional Review Board of Chungnam National University (No. 202107-SB-125-01). Written informed consent was obtained from the participants. However, the opinions are the authors’ alone.

### Study design

This study used structural equation modeling to construct a hypothetical model explaining QoL in high-risk pregnant women based on Roy’s adaptation model [17] and previous research. The study is described according to the STROBE (Strengthening the Reporting of Observational Studies in Epidemiology) reporting guidelines (http://www.strobe-statement.org).

### Participants

The selection criteria for this study were primiparous women who were at least 35 years old (advanced maternal age), living with their spouse, had a gestational age between 20 weeks and 37 weeks, and were diagnosed with a high-risk pregnancy by a specialist. The high-risk pregnancy conditions included 19 specific diseases [25]: preterm labor, postpartum hemorrhage, preeclampsia, premature rupture of membranes, placental abruption, placenta previa, threatened abortion, polyhydramnios, oligohydramnios, antepartum hemorrhage, incompetent internal os of the cervix, pregnancy-induced hypertension, multiple pregnancies, gestational diabetes mellitus, hyperemesis gravidarum, renal disease, heart failure, intrauterine growth restriction, and diseases of the uterus and its appendages. Participants were excluded if they had been diagnosed with cancer or heart disease prior to pregnancy or were currently taking medication for depression. The sample size for this study was determined based on the requirement that 10 to 20 times the number of observed variables is needed for model validation [26]. Given that there were 20 observed variables in this case, a sample size of at least 300 participants was required. To account for a potential 20% dropout rate, a total of 370 participants were recruited [27]. After excluding 37 cases (10%) due to unreliable responses, the final study population consisted of 333 participants (100 recruited in person and 233 recruited online), thereby meeting the aforementioned sample size requirements.

### Study tools

Permission to use the measurement tool was obtained through email communication with the tool developers and the authors of the Korean translation before data collection.

### Adaptation level: *Quality of life*

The Maternal Postpartum Quality of Life Questionnaire (MAPP-QOL), originally developed by Hill and Aldag [28], and later translated into Korean by Choi et al. [29], was adapted by our research team to better suit the characteristics of pregnant women. Despite its initial design for postpartum mothers, the questionnaire’s items were found to be relevant to pregnant women, making it an appropriate tool for assessing their QoL. The original 40-item MAPP-QOL comprises five domains: psychological/baby (eight items), socioeconomic (nine items), relational/spouse-partner (five items), relational/family-friends (10 items), and health and functioning (eight items). Modification involved excluding four items specific to postpartum mothers’ experiences: “in the care of the cesarean section or episiotomy site,” “in the assistance with caring for newborns or other children,” “in the time spent with children,” and “in your ability to breastfeed your child.” This modified version underwent a content validity evaluation by three nursing professors and one obstetric nurse. Using a 4-point scale (4, very valid to 1, not valid at all), all items, except one related to “economic ability” with a content validity index below 0.8, were confirmed to have a validity index of 1.0. Subsequently, the modified MAPP-QOL consisted of 35 items across five domains: psychological/baby (eight items), socioeconomic (eight items), relational/spouse-partner (five items), relational/family-friends (seven items), and health and functioning (seven items). The MAPP-QOL assesses the satisfaction and importance of each item on a scale from 1 to 6. According to the scoring method, the total score and subdomain score ranges are calculated, with scores ranging from a minimum of 0 to a maximum of 30 points. A higher score indicates a higher QoL in pregnant women. During its development, the tool demonstrated reliability with a Cronbach’s ⍺ of .96 [28]; and in this study, the reliability was shown by a Cronbach’s α of .95. The Cronbach’s α values for each subfactor were as follows: psychological/baby, .86; socioeconomic, .87; relational/spouse-partner, .88; relational/family-friends, .85; and health & functioning, .86.

### Contextual stimuli: *Uncertainty*

Mishel’s Uncertainty in Illness Scale [30], which was translated into Korean by Chung et al. [31], was used. This 33-item instrument has four subdomains: ambiguity (13 items), complexity (seven items), inconsistency (seven items), and unpredictability (five items); and an additional item that does not fall within these four subdomains. The scale uses a self-report 5-point Likert scale (1, not at all to 5, very much) and higher scores (possible range, 33–160) indicate a greater level of uncertainty. Cronbach’s α, as a measure of the tool’s reliability, was .91 at the time of its development [30], and.84 in this study.

### Coping mechanisms: *Coping*

The Korean version [32] of the Cognitive Emotion Regulation Questionnaire (CERQ), a cognitive emotion regulation strategy tool developed by Garnefski et al. [9], was used to measure coping. The CERQ categorizes cognitive coping into nine factors, which are further divided into adaptive coping subfactors, which include putting into perspective, refocusing on planning, acceptance, positive refocusing, and positive reappraisal, and maladaptive coping subfactors, which include self-blame, blaming others, rumination, and catastrophizing. The CERQ consists of 36 items, rated on a 5-point Likert scale (1, almost never, to 5, almost always). Adaptive coping has a possible range of 20 to 100 points, maladaptive coping has a possible range of 16 to 80 points, and each subfactor has a possible range of 4 to 20 points. Higher subfactor scores indicate a higher usage of cognitive strategies. The reliability of the tool, as measured by Cronbach’s α, was .80 at the time of its development [33] and .86 in this study. The reliability of the subfactors was as follows: putting into perspective, .72; refocusing on planning, .82; acceptance, .62; positive refocusing, .83; positive reappraisal, .78; self-blame, .80; blaming others, .84; rumination, .72; and catastrophizing, .71.

### Adaptation modes

#### Fatigue

This study utilized a score derived from a simplified 10-item fatigue scale. This scale, originally developed by Milligan et al. [34], was later translated into Korean, modified, and revised by Song [35]. The tool consists of physical and mental dimensions, each rated on a 4-point Likert scale (1, not at all, to 4, very much). A higher score (possible range, 10–40) signifies a higher level of fatigue. In Song’s study [35], the Cronbach’s α value was .88, while in this study, it was .86.

#### State anxiety

State anxiety was assessed using the State-Trait Anxiety Inventory [36], which was translated and validated in Korean [37]. The inventory comprises 20 items, each rated on a 4-point Likert scale (1, not at all, to 4, very much). A higher score (possible range, 20–80) indicates a greater level of state anxiety. The reliability of the inventory was good during its initial development, i.e., Cronbach’s α value of .92 [36], as well as in this study .92.

#### Antenatal depression

The Korean version [38] of the Edinburgh Postnatal Depression Scale (EPDS) [39] was utilized to assess antenatal depression which has been confirmed as reliable and valid for antenatal depression as well. The 10-item EPDS assesses depression, anxiety, fear, guilt, and suicidal thoughts. The total score ranges from 0 to 30 points and a cutoff score of 9/10 is used for Korean women, with scores above 10 indicating a higher degree of antenatal depression [38]. The reliability of the Korean version was good, i.e., Cronbach’s α value of .87 in a prior study [38], and.81 in this study.

#### Maternal identity

Maternal identity scores were derived using a 40-item instrument developed by Kim and Hong [40]. Twenty items each assess behavioral factors and emotional factors. Each item is rated on a 4-point Likert scale (1, not at all, to 4, very much) and higher scores (possible range, 40–160) suggest a more effective performance of the anticipated maternal role, enhanced interaction between the expectant mother and the fetus, and a positive emotional state [40]. The tool’s reliability was good, i.e., Cronbach’s α of .92 at development [40], and .92 in this study.

#### Marital adjustment

The Korean adaptation [41] of the Dyadic Adjustment Scale (DAS) [42], specifically the abbreviated DAS-10 item version, was used to assess discrepancies between spouses, marital satisfaction, and spousal cohesion [41]. Of the total score (possible range, 1–51), a cutoff of 32 points is applied, with higher scores signifying greater marital adjustment. In the study conducted by Cho et al. [41], Cronbach’s α was reported as .83, while in this study, it was found to be .88.

### General and obstetric characteristics

The general characteristics of the participants, such as age, education level, employment status, economic status, and length of marriage, were measured. Obstetric characteristics included gestation period, experience with hospitalization, prenatal education, diagnosis of a high-risk pregnancy, and subjective health status.

### Data collection

Data were collected from October 20, 2021 to February 20, 2022, using both in-person and online methods. The in-person data collection was carried out after explaining the research objectives and securing approval from the directors and nursing staff of two obstetrics and gynecology departments. Posters were displayed in outpatient departments, and the survey took approximately 20 to 30 minutes to complete. Upon completion, each participant placed their questionnaire in a sealed envelope. The collected data were then coded, inputted, and stored in password-protected files. For online data collection, cooperation was obtained from the administrators of a large online community for pregnant women in Korea, known as ‘MomsHolic.’ The researcher posted recruitment posters on the site, and participants who were interested could express their willingness to participate by clicking on a link provided in the research description, as specified by the research administrator. Individual survey links were then sent to these participants for data collection. All participants in the study received a mobile coupon (worth roughly 4 US dollars) as a token of appreciation.

### Data analysis

The data analysis was carried out using IBM SPSS ver. 26.0 and AMOS version 26.0 (IBM Corp., Armonk, NY, USA). Descriptive statistics, including difference tests, correlations, and reliability were done for the participants’ general characteristics and the variables measured. Cronbach’s α was used to assess the reliability of the research instruments. To validate construct validity, model fit, total effects among variables, direct effects, indirect effects, and explanatory power as a structural equation model, we performed exploratory factor analysis and confirmatory factor analysis using the AMOS program. We assessed the normality of the sample through skewness and kurtosis. To check for multicollinearity among the measurement variables, we examined tolerance, variance inflation factor, and Pearson correlation coefficients. The estimation for the structural model assumed multivariate normality and utilized maximum likelihood estimation. We assessed the fit of the hypothesis model using χ^2^, χ^2^/df, goodness of fit index (GFI), standardized root mean square residual (SRMR), root mean square error of approximation (RMSEA), comparative fit index (CFI), the Tucker-Lewis index (TLI), and parsimonious normed fit index (PNFI). To verify the statistical significance of the research model, we used bootstrapping (1,000 iterations) to test the significance of total effects, direct effects, and indirect effects.

## Results

### Differences in quality of life according to participants’ characteristics

The mean age of the study participants was 35.29 (±3.72) years, ranging from 26 to 45 years, and the majority (51.1%) were under 35 years old (n=170). Most participants had a college degree (n=299, 89.8%), 58.6% (n=195) reported not having a job, and 39.0% (n=130) had an income of over 6 million Korean won. The mean duration of marriage was 40.33 (±28.01) months, ranging from 3 to 180 months. The participants’ gestational period averaged 28.75 (±4.74) weeks, with 50.5% (n=168) between 20 and 28 weeks and 49.5% (n=165) between 29 and 37 weeks. Among the participants, 35.4% (n=118) reported a history of hospitalization, and 38.1% (n=127) received antenatal education. Regarding high-risk pregnancy diagnoses, 48.6% (n=162) were first-time mothers over 35 years old, followed by 26.1% (n=87) with gestational diabetes mellitus and 21.6% (n=72) with preterm labor. Self-reported health status was perceived as poor by 18.3% (n=61) and average by 46.3% (n=154) (Table 1).

Significant differences were observed in the QoL scores based on participant characteristics such as education level, employment status, gestational period, and self-reported health status. Participants who held a college degree demonstrated significantly higher QoL scores (t=–2.53, *p*=.012). Similarly, those who were employed also had significantly higher scores compared to those who were not (t=2.92, *p*=.004). Participants at 20 to 28 weeks of gestation had higher QoL scores (t=2.50, *p*=.013). Furthermore, participants who reported a good subjective health status had significantly higher QoL scores (F=16.89, *p*<.001) (Table 1).

### Descriptive statistics and verification of the study variables’ validity

The mean total score for QoL was 18.63 (±3.80), indicating an above-average level. The subscale scores were as follows: psychological/baby, 19.03 (±4.48); socioeconomic, 19.00 (±4.60); relational/spouse-partner, 20.99 (±4.58); relational/family-friends, 19.18 (±4.78); and health & functioning, 16.18 (±4.19). Among these, the relational/spouse-partner subscale had the highest score, while health & functioning had the lowest.

Uncertainty had a mean score of 91.60 (±14.29), indicating an above-average level. Adaptive coping had a mean score of 79.39 (±10.31), with the following sub-scores: perspective scoring, 15.70 (±2.73); refocus on planning, 16.73 (±2.34); acceptance, 15.88 (±2.22); positive refocusing, 14.94 (±3.06); and positive reappraisal, 16.14 (±2.52). Maladaptive coping had a mean score of 47.32 (±9.63), with the following sub-scores: self-blame, 12.68 (±2.74); blaming others, 9.72 (±3.38); rumination, 13.23 (±3.20); and catastrophizing, 11.69 (±3.31). Fatigue had a mean score of 27.67 (±5.73), state anxiety had a mean score of 44.65 (±10.49), and antenatal depression had a mean score of 10.54 (±5.11), with 57.6% (n=192) scoring 10 or higher. Maternal identity had a mean score of 126.51 (±16.35), and marital adjustment had a mean score of 38.19 (±6.10), both indicating above-average levels (Supplementary Table 1).

The correlation coefficient values between the measured variables ranged from *r*=–.01 to .75, suggesting no issues with multicollinearity (*r*>±.90). The variance inflation factors varied from 1.51 to 3.84, all of which were below 10, further indicating no multicollinearity between the measured variables. The average variance extracted for the latent factors in this study ranged from .61 to .94, all-surpassing 0.5, and the composite construct reliability exceeded 0.6, thereby confirming both convergent and discriminant validity (Supplementary Table 1). Upon examining the assumption of multivariate normality for the structural equation model, a multivariate kurtosis index of 69.092 was found, which violated the normality assumption. As a result, the most commonly used maximum likelihood estimation was selected for parameter estimation, and bootstrapping, a beneficial method for analyzing data that deviates from multivariate norms, was chosen.

### Verification of the fit of the hypothetical model

#### Results of the testing and modification of the hypothetical model

The test results of the hypothetical model revealed that the absolute fit indices (χ^2^=405.07, χ^2^/df=3.94, GFI=.90, SRMR=.11, RMSEA=.09), the incremental fit indices (CFI=.92 and TLI=.90), and the parsimonious fit index (PNFI=.90), did not fully satisfy the recommended criteria for absolute fit indices—specifically, this was the case for χ^2^, χ^2^/df, SRMR, and RMSEA. To improve the model fit, we conducted explorations of the relationships between variables and their theoretical foundations. Drawing on previous research that suggests a direct impact of coping on QoL [43], we added two paths to the hypothetical model: one from adaptive coping to QoL, and another from maladaptive coping to QoL. The final modified model showed adequate absolute fit indices (χ^2^=261.11 [<.001], χ^2^/df=2.69, GFI=.93, SRMR=.05, and RMSEA=.07), incremental fit indices (CFI=.95 and TLI=.91), and parsimonious fit index (PNFI=.47). These results met the adequacy criteria for both the absolute fit indices and the incremental fit indices (Table 2).

#### Results of the effect analysis in the modified model

In the modified model’s estimated paths, six out of seven total paths were found to be statistically significant. The coping model revealed significant paths from uncertainty to both adaptive coping (β=–.26, *p*=.006) and maladaptive coping (β=.68, *p*=.014). Similarly, in the adaptation mode model, both adaptive coping (β=–.44, *p*=.018) and maladaptive coping (β=.69, *p*=.012) demonstrated significant paths. Lastly, in the final QoL model, the adaptation mode (β=–.81, *p*=.034) and maladaptive coping (β=.46, *p*=.043) were identified as significant paths.

**Figure 3.**
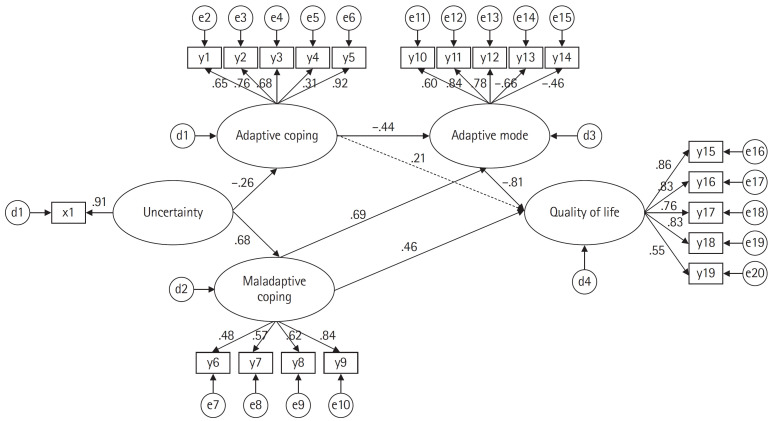
Path diagram of the modified model. x1: uncertainty; y1: putting into perspective; y2: refocus in planning; y3: acceptance; y4: positive refocusing; y5: positive reappraisal; y6: self-blame; y7: blaming others; y8: rumination; y9: catastrophizing y10: fatigue; y11: state anxiety; y12: antenatal depression; y13: maternal identity; y14: marital adjustment; y15: psychological/baby; y16: socioeconomic; y17: relational/spouse-partner; y18: relational/family-friends; y19: health and functioning.

The variable that most significantly influenced the QoL in high-risk pregnant women was the adaptation mode. Both direct and indirect effects were significantly demonstrated by maladaptive coping, while uncertainty showed a significant indirect effect. These factors had an explanatory power of 51%. The variable that had the most profound impact on the adaptation mode in high-risk pregnant women was maladaptive coping. Maladaptive coping displayed a significant direct effect, whereas uncertainty showed a significant indirect effect. These factors exhibited an explanatory power of 79%. Uncertainty in high-risk pregnant women significantly directly affected both adaptive and maladaptive coping. Adaptive coping had an explanatory power of 7%, while that of maladaptive coping was 47% (Figure 3, Table 3).

## Discussion

This study constructed a hypothetical model based on Roy’s adaptation theory [17] and informed by concepts from literature reviews, to elucidate the QoL in high-risk pregnant women. We then tested the model’s adequacy and the significance of its pathways using a sample of 333 high-risk pregnant women. Factors that explained the QoL demonstrated direct effects for adaptation mode and maladaptive coping, and indirect effects for uncertainty, adaptive coping, and maladaptive coping. The results of this study prompt a discussion on variables associated with the QoL in high-risk pregnant women and the implications for their nursing care.

Of the primiparous women diagnosed with high-risk pregnancies, their. Approximately half of these high-risk pregnant women were over 35 years old, which is considered advanced maternal age. In 2018, the rate of advanced maternal age pregnancies in Korea was reported to be 31.8% [3]. The higher rate in this study may be due to the deliberate self-selection of high-risk pregnant women of advanced maternal age. Preterm labor is often reported as a common health issue in high-risk pregnancies [4]. The high incidence of gestational diabetes mellitus in this study is likely due to the fact that the participants were recruited from outpatient obstetrics and gynecology clinics. Among the participants, 118 (35.4%) had a history of hospitalization, which reflects the efforts of high-risk pregnant women to prevent adverse outcomes related to preterm labor [6]. However, this could also contribute to an increased burden of pregnancy and uncertainty about the prognosis compared to women with low-risk pregnancies. The percentage of participants who reported poor subjective health status was 18.3%, which is slightly higher than the 16.8% reported for hospitalized high-risk pregnant women [44] and similar to the 18.4% reported for high-risk pregnant women receiving outpatient care [45]. When compared to the 15.4% reported in a study on women with low-risk pregnancies [46], it is clear that women diagnosed with high-risk pregnancies tend to perceive their health status more negatively, regardless of whether they are receiving outpatient or inpatient treatment.

The results of this study revealed that the QoL score for high-risk pregnant women averaged 18.63 out of 30 points. This score is comparable to the 18.94 average score of participants who were hospitalized due to preterm labor [14]. Although it was difficult to find studies using the same tool for direct comparison with low-risk pregnant women, the score was lower than that of mothers without prenatal complications, who averaged 19.64 points [47]. This suggests that the QoL for high-risk pregnant women may be lower than that for low-risk pregnant women. This conclusion aligns with the findings of systematic literature review studies [48], which indicate that the QoL for high-risk pregnant women is indeed lower compared to their low-risk counterparts. These results underscore the necessity for medical care and intervention strategies that are specifically designed for the unique circumstances of high-risk pregnant women, going beyond standard therapeutic interventions and health maintenance.

Although the initial hypothetical model did not meet the recommended standards, modifications were made to confirm the final model. This revised model achieved the recommended levels with a chi-square to degrees of freedom ratio of 2.69, and both SRMR and RMSEA were below 0.08. GFI, CFI, and TLI values all exceeded 0.90, indicating a good fit [26]. The modified model demonstrated that factors such as uncertainty, adaptive coping, maladaptive coping, and adaptation mode in high-risk pregnant women accounted for their QoL. Conversely, a structural model study on the health-related QoL in low-risk pregnant women [49] identified sleep quality, physical activity, and perceived health status as explanatory factors. This highlights the differences in factors that explain the QoL in pregnant women, depending on their risk status.

The uncertainty score for participants in this study (91.60 points) was comparable to the score of 97.31 observed in pregnant women hospitalized due to high-risk pregnancies [50]. Given that the participants in this study were diagnosed with high-risk pregnancies and were receiving both outpatient and inpatient care, the heightened uncertainty can likely be attributed to their high-risk pregnancy diagnosis. This study reinforces the idea that uncertainty influences coping strategies, leading to a decrease in adaptive coping and an increase in maladaptive coping [7]. However, it was observed in this study that high-risk pregnant women tended to rely more on maladaptive coping than adaptive coping to manage the negative emotions triggered by the high-stress situation of a high-risk pregnancy. This observation is consistent with research that suggests an increase in uncertainty leads to a decrease in adaptive coping and an increase in maladaptive coping [51]. Moreover, high-risk pregnant women perceived uncertainty as contextual stimuli, which negatively affected their QoL. This finding is in line with research that proposes high levels of uncertainty can cause high-risk pregnant women to harbor negative thoughts about their lives, making it challenging for them to actively cope, and potentially leading to maladaptive outcomes during pregnancy [22].

The adaptation mode of the participants in this study was analyzed in terms of fatigue, state anxiety, antenatal depression, maternal identity, and marital adjustment. The fatigue score (27.67 points) was comparable to the score of 27.78 points observed in low-risk pregnant women during the later stages of pregnancy [52]. However, the state anxiety score for high-risk pregnant women (44.62 points) was 1.5 times higher than the score of 29.20 points seen in low-risk pregnant women [53]. Moreover, the antenatal depression score was 10.54 points, 1.7 times higher than the score of 6.12 points for low-risk pregnant women [54], suggesting the presence of mild depressive symptoms. The maternal identity score was 126.51 points, lower than the score of 131.15 points for low-risk pregnant women [55] and comparable to the score of 127.80 points for pregnant women with gestational diabetes mellitus [56]. This implies that high-risk pregnant women may face challenges in attachment behavior and transitioning to motherhood compared to their low-risk counterparts. The marital adjustment score (38.19 points) was lower than the score of 41.06 points for low-risk pregnant women [54], suggesting less stability and satisfaction in the marital lives of high-risk pregnant women. If marital relationships are unsatisfactory, it may lead to negative emotions in pregnant women and adversely affect their QoL. Therefore, it is important to understand and consider the aspect of marital adjustment in high-risk pregnant women.

Upon examining the factors in the model, it was observed that uncertainty in high-risk pregnant women indirectly impacted their QoL. A study on breast cancer patients reported a significant indirect effect [57], but additional repetitive research is required to confirm the indirect factors of uncertainty that affect the QoL in high-risk pregnant women. Adaptive coping demonstrated a significant indirect effect, while maladaptive coping was found to have significant direct and indirect effects on the QoL. Considering that adaptive coping is employed to effectively manage physical and emotional well-being, providing information on stress management techniques and high-risk pregnancy could assist in promoting adaptive coping strategies [7]. Maladaptive coping, a strategy often used by high-risk pregnant women [51], can intensify negative psychological issues such as anxiety and depression, and hinder the transition to motherhood. Therefore, it is vital to help these women reduce their reliance on such strategies. The adaptation mode was found to have a significant direct effect on the QoL. In this study, the adaptation mode, which includes physiological indicators like fatigue and self-concept indicators such as anxiety and depression, showed a negative correlation with the QoL. Role function indicators like maternal identity and interdependence indicators such as marital adjustment also exhibited a static correlation with the QoL. These findings suggest that the adaptation mode of high-risk pregnant women operates in a mutually related manner, exerting a negative direct effect on the QoL and thus reducing it. This highlights the necessity for a comprehensive perspective on how individuals adapt to various stimuli in their lives.

Although the participants experienced high levels of uncertainty, fatigue, anxiety, and depression, their QoL remained above average. This can be attributed to the positive indirect effect of adaptive coping strategies, which were mediated by the adaptation mode. Additionally, the direct effect of maladaptive coping strategies also influenced QoL. This finding is consistent with previous research suggesting that the QoL in high-risk pregnant women is significantly influenced by their coping strategies [12]. Consequently, it is recommended that nursing interventions be planned to enhance adaptive coping and reduce maladaptive coping strategies, as this could improve QoL for high-risk pregnant women.

This study has several limitations, including the use of both in-person and non-in-person data collection methods. The in-person data collection was restricted to outpatient women in a single region, who were recruited through convenience sampling. As such, care should be taken when extrapolating the results of this study to all high-risk pregnant women. The QoL was found to be lower in participants who were high school graduates, unemployed, between 29 and 37 weeks of gestation, and those who reported poor subjective health. However, these factors were not included in the model, so caution is necessary when interpreting the research results. High-risk pregnant women have varying risk factors depending on the type of complication and gestational period. Therefore, it is crucial to analyze changes and causal relationships over time among the various factors that affect QoL. We recommend conducting follow-up studies using longitudinal research to verify the model’s effectiveness in determining the time series effects on QoL throughout pregnancy.

In conclusion, this study provided foundational data for the development of nursing interventions aimed at enhancing the QoL for high-risk pregnant women, drawing on Roy’s adaptation theory. It takes into account a range of factors—physical, psychological, social, and environmental—that could potentially impact the QoL of these women. The study identifies significant direct and indirect pathways among factors related to QoL, underscoring the crucial role of uncertainty management in nursing interventions. It also highlights the importance of encouraging adaptive coping strategies and minimizing the use of maladaptive ones, to help high-risk pregnant women adapt and improve their QoL.

As findings established the influence of coping mechanisms on QoL in high-risk pregnant women, ongoing education and counseling are essential in clinical environments to help these women adjust to pregnancy and employ adaptive coping strategies, rather than resorting to maladaptive ones. For those high-risk pregnant women who exhibit a low capacity for adaptive coping or a propensity to over-rely on maladaptive coping, the implementation of cognitive-behavioral interventions could enhance their QoL and facilitate their adjustment to pregnancy.

## Figures and Tables

**Table 1.** Differences in quality of life according to participants’ characteristics (N=333)

Characteristic	Categories	n (%)	Quality of life	
			Mean ± SD	t/F(p)Scheffé
Age (year)	Mean±SD, 35.29±3.72 (range, 26-45)			
	≤35	170 (51.1)	18.90±3.55	1.01 (.364)
	36–39	118 (35.4)	18.43±3.93	
	≥40	45 (13.5)	18.12±4.37	
Education	High school	34 (10.2)	17.07±2.88	–2.53 (.012)
	College	299 (89.8)	18.80±3.86	
Occupation	Yes	138 (41.4)	19.34±3.90	2.92 (.004)
	No	195 (58.6)	18.12±3.66	
Monthly compensation (million KRW)	<2	5 (1.5)	18.13±0.94	0.11 (.956)
	2–3.9	82 (24.6)	18.55±3.68	
	4–5.9	116 (34.9)	18.77±4.03	
	≥6	130 (39.0)	18.57±3.77	
Marital duration (month)	3–36	192 (57.7)	18.76±4.05	1.29 (.280)
	37–60	79 (23.7)	18.09±3.33	
	61–180	62 (18.6)	18.90±3.57	
Gestational period (week)	20–28	168 (50.5)	19.14±4.05	2.50 (.013)
	29–37	165 (49.5)	18.10±3.47	
Hospitalization experience	Yes	118 (35.4)	18.66±3.82	0.11 (.917)
	No	215 (64.6)	18.61±3.81	
Prenatal education	Yes	127 (38.1)	18.25±3.35	–1.42 (.158)
	No	206 (61.9)	18.86±4.05	
Classification of high-risk pregnancies^†^	Advanced maternal age^‡^	162 (48.6)	18.30±4.05	
	Gestational diabetes mellitus	87 (26.1)	18.42±3.97	
	Preterm labor	72 (21.6)	19.17±3.62	
	Incompetent internal os of cervix	29 (8.7)	17.54±4.30	
	Pregnancy-induced hypertension	29 (8.7)	20.80±2.59	
	Multiple pregnancies	29 (8.7)	19.90±4.23	
	Antepartum hemorrhage	21 (6.3)	18.93±2.24	
	Placenta previa	18 (5.4)	19.54±2.87	
	Preeclampsia	16 (4.8)	17.63±3.88	
	Hyperemesis gravidarum	13 (3.9)	17.13±2.60	
	Premature rupture of membrane	8 (2.4)	15.39±1.87	
	Oligohydramnios	8 (2.4)	15.50±2.20	
Perceived health status	Poor^a^	61 (18.3)	17.26±3.62	16.89 (<.001) (a,b<c)
	Moderate^b^	154 (46.3)	18.00±3.72	
	Good^c^	118 (35.4)	20.15±3.53	

KRW: Korean won (1 million KRW is approximately 800 US dollars).

† Multiple responses.

‡ ≥35 years.

**Table 2.** Model fit measures of the preliminary and modified models

Model	χ^2^	χ^2^/df	GFI	SRMR	RMSEA	CFI	TLI	PNFI
Model criteria	*p*>.05	≤3.00	≥.90	≤.08	≤.08	≥.90	≥.90	≥.60
Hypothetical model	405.07 (<.001)	3.94	.90	.11	.09	.92	.90	.90
Modified model	261.11 (<.001)	2.69	.93	.05	.07	.95	.91	.47

CFI: comparative fit index; GFI: goodness of fit index; PNFI: parsimonious normed fit index; RMSEA: root mean square error of approximation; SRMR: standardized root mean square residual; TLI: Tucker-Lewis index.

**Table 3.** Standardized estimates, standardized direct, indirect, and total effects for the modified hypothetical model (N=333)

Endogenous variable	Exogenous variable	SMC	Standardized effect, β (*p*)		
			Direct	Indirect	Total
Adaptive coping	Uncertainty	.07	–.26 (.006)		–.26 (.006)
Maladaptive coping	Uncertainty	.47	.68 (.014)		.68 (.014)
Adaptation mode	Adaptive coping	.79	–.44 (.018)		–.44 (.018)
	Maladaptive coping		.69 (.012)		.69 (.012)
	Uncertainty			.58 (.023)	.58 (.023)
Quality of life	Adaptation mode	.51	–.81 (.034)		–.81 (.034)
	Adaptive coping		.21 (.119)	.36 (.026)	.57 (.005)
	Maladaptive coping		.46 (.043)	–.56 (.023)	–.10 (.027)
	Uncertainty			–.21 (.004)	–.21 (.004)

SMC, squared multiple correlation; β, standardized coefficient.

## Supplementary materials

Further details on supplementary materials are presented online (available at https://doi.org/10.4069/kjwhn.2023.11.13.1).
