# Supplementary material for: An explanatory model of quality of life in high-risk pregnant women in Korea: a structural equation model
Source: Korean J Women Health Nurs. 2023 Dec 28;29(4):302–16. doi: 10.4069/kjwhn.2023.11.13.1 (PMC10788389; doi:10.4069/kjwhn.2023.11.13.1)
Supplement: Supplementary Table 1. [file kjwhn-2023-11-13-1-Supplementary-Table-1.pdf]

Supplementary Table 1. Descriptive statistics and relationships among measured variables (N=333)

Variable	1	2	3	4	5	6	7	8	9	10	11	12	13	14	15	16	17	18	19	20
1. Uncertainty	1																			
2. Putting into perspective	–.19***	1																		
3. Refocus in planning	–.18***	.48***	1																	
4. Acceptance	–.11*	.56***	.66***	1																
5. Positive refocusing	0.01	.52***	.46***	.34***	1															
6. Positive reappraisal	–.16**	.61***	.73***	.66***	.59***	1														
7. Self-blame	.28***	0.06	0.03	.17**	–.02	0.07	1													
8. Blaming others	.40***	–.07	–.11*	–.11*	.16**	–.06	.30***	1												
9. Rumination	.30***	–.04	.18***	.22***	0.03	0.11	.42***	.32***	1											
10. Catastrophizing	.48***	–.14*	–.10	0.03	–.05	–.14**	.50***	.48***	.60***	1										
11. Fatigue	.47***	0.01	–.01	0.08	0.09	–.08	.17**	.22***	.31***	.40***	1									
12. State anxiety	.46***	–.36***	–.37***	–.20***	–.29***	–.39***	.27***	.30***	.33***	.57***	.38***	1								
13. Antenatal depression	.56***	–.29***	–.29***	–.19***	–.12*	–.33***	.30***	.33***	.24***	.50***	.32***	.68***	1							
14. Maternal identity	.12*	.38***	.44***	.34***	.36***	.41***	.14**	.14*	.22***	.16**	.27***	–.09	0.01	1						
15. Marital adjustment	–.30***	.32***	.38***	.25***	.22***	.41***	–.12*	–.33***	–.17**	–.29***	–.18***	–.46***	–.47***	.25***	1					
16. Psychological/baby	–.23***	.45***	.40***	.29***	.50***	.49***	–.02	0.02	–.1	–.22***	–.17**	–.64***	–.43***	.31***	.38***	1				
17. Socioeconomic	–.06	.29***	.46***	.32***	.43***	.45***	0.01	0.05	0.08	–.03	–.1	–.36***	–.24***	.35***	.27***	.62***	1			
18. Relational/ spouse-partner	–.16**	.34***	.40***	.28***	.31***	.44***	–.06	–.11*	–.11*	–.23***	–.16**	–.43***	–.34***	.40***	.60***	.62***	.63***	1		
19. Relational/ family-friends	–.12*	.31***	.37***	.27***	.40***	.45***	0.01	0.06	–.03	–.15**	–.12*	–.45***	–.30***	.37***	.35***	.72***	.71***	.75***	1	
20. Health and functioning	–.13*	.38***	.27***	.19***	.43***	.33***	.13*	.23***	–.08	–.04	–.11*	–.33***	–.19***	.20***	0.08	.72***	.54***	.36***	.56***	1
Mean ± SD	91.60 ± 14.29	15.70 ± 2.73	16.73 ± 2.34	15.88 ± 2.22	14.94 ± 3.06	16.14 ± 2.52	12.68 ± 2.74	9.72 ± 3.38	13.23 ± 3.20	11.69 ± 3.31	27.67 ± 5.73	44.65 ± 10.49	10.54 ± 5.11	126.51 ± 16.35	38.19 ± 6.10	19.03 ± 4.48	19.00 ± 4.60	20.99 ± 4.58	19.18 ± 4.78	16.18 ± 4.19
Skewness	–.46	–.13	–.45	–.03	–.35	–.29	0.14	0.59	–.45	0.3	–.02	–.13	0.01	–.25	–59	–.58	–.02	–.52	–.03	0.15
Kurtosis	–.49	–.77	0.07	–.18	–.04	–.19	–.37	–.41	–.18	–.37	–.65	–.28	–.66	–.73	–.52	1.16	–.16	0.92	0.08	0.05
AVE		0.9					0.79				0.61					0.94				
CR		0.97					0.94				0.6					0.99				

AVE, average variance extracted; CR, construct reliability.
**p*<.05, ***p*<.01, ****p*<.001.

https://doi.org/10.4069/kjwhn.2023.11.13.1
